# Impact of Pyrethroid Plus Piperonyl Butoxide Synergist-Treated Nets on Malaria Incidence 24 Months after a National Distribution Campaign in Rwanda

**DOI:** 10.4269/ajtmh.23-0317

**Published:** 2023-10-23

**Authors:** Michee Kabera, Jean-Louis N. Mangala, Radina Soebiyanto, Beata Mukarugwiro, Kaendi Munguti, Aimable Mbituyumuremyi, Naomi W. Lucchi, Emmanuel Hakizimana

**Affiliations:** ^1^ Malaria and Other Parasitic Diseases Division, Rwanda Biomedical Centre, Kigali, Rwanda;; ^2^ U.S. President’s Malaria Initiative, U.S. Agency for International Development (USAID), Washington, District of Columbia;; ^3^ U.S. President’s Malaria Initiative, USAID, Kigali, Rwanda;; ^4^ U.S. President’s Malaria Initiative, U.S. Centers for Disease Control and Prevention, Kigali, Rwanda

## Abstract

Malaria remains a public health priority in Rwanda. The use of insecticide-treated nets (ITNs) is a key malaria prevention tool. However, expanding pyrethroid resistance threatens the gains made in malaria control. In 2018, the Rwandan malaria program strategic approach included the use of newer types of ITNs such as pyrethroid plus piperonyl butoxide (PBO) synergist-treated nets to counter pyrethroid resistance. In February 2020, 5,892,280 ITNs were distributed countrywide; 1,085,517 of these were PBO nets distributed in five districts. This study was a pragmatic observational study that leveraged the 2020 net distribution and routinely collected confirmed malaria cases to determine the impact of PBO nets 1 and 2 years after ITN distribution. No differences were observed in the average net coverage between the PBO and standard net districts. A significant reduction in malaria incidence was reported in both the PBO (*P* = 0.019) and two control districts that received standard nets (*P* = 0.008) 1 year after ITN distribution. However, 2 years after, this reduction was sustained only in the PBO (*P* = 0.013) and not in the standard net districts (*P* = 0.685). One year after net distribution, all districts had a significant reduction in malaria incidence rate (incidence rate ratio < 1). In the second year, incidence in districts with PBO nets continued to decrease, whereas in districts with standard nets, incidences were similar to predistribution levels. The results indicate that PBO nets are a promising tool to combat pyrethroid resistance in Rwanda, with protective effects of up to 2 years post distribution.

## INTRODUCTION

With an estimated 247 million cases and 619,000 deaths reported in 2021 globally,^1^ malaria continues to be a global public health problem, especially in Africa, which accounted for 95% (228 million) of all malaria cases and deaths in 2021.^1^ In Rwanda, the entire population of about 12.9 million is at risk of malaria infection, although a high burden of malaria is mainly observed in certain districts.^2^ There are four main malaria epidemiological zones based on the 2016 annual parasite incidence (API; number of confirmed new cases [febrile illness plus parasitemia] measured in all ages, expressed per 1,000 individuals under surveillance) per district: high endemicity zone: > 450 API per 1,000 persons; moderate endemicity zone: 250–450 API per 1,000 persons; low endemicity zone: 100–250 API per 1,000 persons; and very low endemicity zone: < 100 API per 1,000 persons (Rwanda Malaria National Strategic Plan, 2020–2024). The country experienced a resurgence of malaria between 2013 and 2017, which was attributed to many factors, including pyrethroid resistance, climatic changes, increased water bodies, and irrigation schemes as a result of development projects such as rice fields, shifts in mosquito behavior to earlier night and outdoor biting, insufficient coverage of vector control interventions, and increased case detection and reporting. However, recent data indicate that malaria is declining, with the incidence of malaria in Rwanda lessening from 321 per 1,000 persons per year in 2018–2019 to 96 per 1,000 persons per year in 2021–2022 after the scaling up and sustaining of several interventions by the malaria control program.

Malaria is transmitted by the bite of an infected anopheline mosquito, and as such, vector control activities are important components of prevention efforts. The main vector control measures used globally by many national malaria control programs are insecticide-treated nets (ITNs) and indoor residual spraying (IRS). Aligned with these core vector control interventions, the Rwandan malaria program adopted similar strategies to control malaria with universal coverage with ITNs and IRS in 12 high malaria burden districts. Both ITNs and IRS depend on the use of an effective insecticide and a high coverage in usage for successful impact.^3^ The most widely used insecticides in Rwanda for ITNs were pyrethroid based (mainly deltamethrin, permethrin, and lambdacyhalothrin), and for many years, nonpyrethroid insecticide for IRS, from 2013. However, studies in many countries including Rwanda have confirmed the development of resistance by mosquitoes to pyrethroids.^4^ Entomological evidence suggests that pyrethroid nets are less effective at killing mosquitoes under household conditions,^5^^,^^6^ and pyrethroid resistance was shown to contribute to control failure in some countries.^4^^,^^7^ The use of effective insecticides to which the vectors are susceptible has been shown to restore malaria control efforts; for example, IRS results with the use of effective insecticides were shown to lead to restoration of malaria control.^8^^,^^9^

The WHO has encouraged the development of new types of insecticides for use in ITNs and IRS in efforts to counter the failure of current control tools due to insecticide resistance.^10^ One of these is the long-lasting insecticidal net that uses the synergist piperonyl butoxide (PBO) in addition to pyrethroid. PBO inhibits mosquito pyrethroid-metabolizing cytochrome P450 enzymes known to inactivate pyrethroids in insecticide-resistant mosquitoes.^11^ Therefore, the inclusion of PBO in pyrethroid-impregnated nets circumvents these mosquito metabolic resistance mechanisms, thereby partially restoring susceptibility to pyrethroid.^7^^,^^12^^,^^13^ Such PBO pyrethroid-treated ITNs were shown to have similar or better efficacy against resistant mosquitoes under controlled household conditions than the traditional standard ITNs.^14^^,^^15^ The WHO expert group reviewing the evidence for pyrethroid plus PBO nets approved their deployment, albeit in a limited way with a small rollout in specific situations.^16^ Recent community randomized controlled trials conducted in Tanzania^7^ and Uganda^17^^–^^19^ and results reviewed by Gleave et al.^20^ have shown that PBO nets are a promising new tool to reduce the impact of pyrethroid resistance.

In Rwanda, pyrethroid resistance was observed in mosquitoes collected from most of the country’s 12 sentinel sites from 2011, and this was shown to be on the rise in samples collected in 2013.^21^ Resistance was measured by rearing collected *Anopheles gambiae *sensu lato larvae to adults and analyzing them for knockdown and mortality using WHO insecticide test papers with standard diagnostic doses of the recommended insecticides. Susceptibility was restored by the use of PBO, which implied a metabolic mechanism of resistance.^21^ In 30 sites surveyed in 2021, pyrethroid insecticide resistance was confirmed in 37, 83, and 56% of the sites tested for 0.05% deltamethrin, 0.75% permethrin, and 0.05% alphacypermethrin, respectively (Rwanda Ministry of Health, Malaria and Neglected Tropical Diseases Annual Report, Fiscal Year 2020–2021).

Insecticide-treated nets in Rwanda are distributed via two main channels: 1) routine distribution to pregnant women and children less than 1 year old during antenatal clinic (ANC) visits and expanded program of immunization (EPI) services, and 2) distribution to households via mass campaigns organized every 2–3 years. In 2020, as a way of responding to insecticide-resistant mosquito populations, the Rwanda malaria control program deployed PBO synergist nets in five districts; dual-insecticide, Interceptor G2, nets were distributed in four districts with moderate malaria burden. Two brands of standard insecticide treated nets were deployed in the nine remaining districts with low to medium malaria burden and 12 high-burden districts receiving IRS. A total of 5,892,280 rectangular ITNs were distributed in all 30 districts, with 93.7% of these via mass campaigns. The remaining 6.3% were via routine distributions to pregnant women and children.

In this study, comparisons in malaria incidences, using routine data, in districts receiving PBO nets and districts receiving standard nets were made at three time points relative to the net distribution: 12 months before net distribution and 12 and 24 months after the distribution.

## MATERIALS AND METHODS

### Study design.

This was a pragmatic observational study that leveraged the 2020 ITN mass distribution campaign and the malaria incidence data collected in the routine health management information system (HMIS). The study used malaria data obtained in the five districts that received the PBO nets and two comparable districts that received standard nets. All seven districts used standard nets prior to the 2020 mass campaign. The primary outcomes were the district-level incidence of malaria assessed 12 months before net distribution and 12 and 24 months after net distribution.

### Net distribution.

The PBO nets (PermaNet 3.0) were treated with a pyrethroid insecticide and PBO synergist. The fabric of the nets consisted of polyester at the side panels, coated with deltamethrin insecticide and strengthened borders, and a polyethylene roof with both deltamethrin and PBO incorporated. The roof was treated with 4 g/kg of deltamethrin and 25 g/kg of PBO. The sides of the bed nets were treated only with deltamethrin at 2.1 g/kg. The standard nets were made of polyester fabric and treated with deltamethrin at 1.85 g/kg of insecticide content. The distribution of both PBO and standard nets was done in February 2020 via a mass campaign from a distribution point in health centers and routine channels according to the integrated malaria control guidelines (National Integrated Malaria Control Guidelines, 2020 edition). This was based on a needs assessment of ITNs from each household according to the total number of household members. The assumption used was one ITN for 1.8 people according to WHO results-based management guidance.^22^ The estimated quantities of ITNs needed per village and district were calculated in advance by decentralized levels with the support of community health workers (CHWs) and then approved by local authorities.

### Study areas.

**Figure 1.**
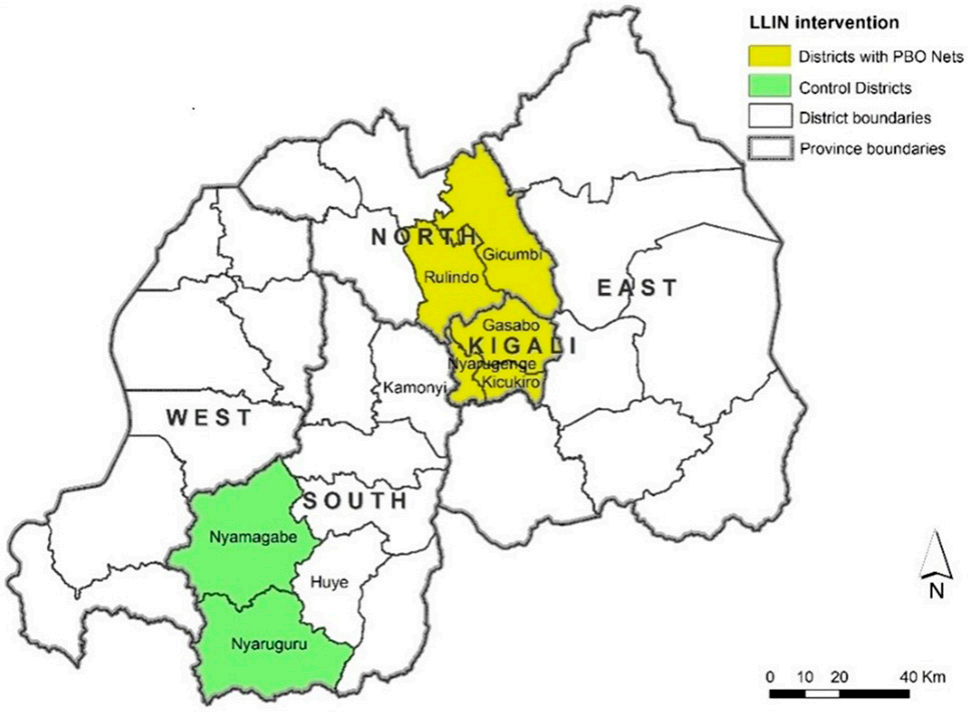
Distribution of PBO and standard nets. LLIN = long-lasting insecticidal net; PBO = pyrethroid plus piperonyl butoxide.

The PBO nets were distributed in five moderate malaria districts with confirmed resistance to pyrethroids: Rulindo and Gicumbi, in the rural Northern Province, and Kicukiro, Gasabo, and Nyarugenge, in Kigali City, as per the Rwanda Malaria Program Distribution Plan, Fiscal Year 2019–2020 (Figure 1). The two standard net districts of Nyamagabe and Nyaruguru are located in the rural southeast of Southern Province with low to moderate malaria with suspected resistance to pyrethroids. The altitude in all districts ranges between 1,300 and 3,000 m of elevation above sea level, with a tropical type of climate characterized by an annual average rainfall ranging from about 950 to 1,500 mm with two annual peaks occurring in April and November. The high dry season is extended from June to September with some sporadic rains. The average temperatures are stable all year round and vary between 16 and 28°C.

The PBO nets were distributed in Rulindo, Gicumbi, Kicukiro, Gasabo, and Nyarugenge (yellow fill) and the standard nets were distributed in Nyamagabe and Nyaruguru districts (green fill) located in the rural southeast of Southern Province.

### Determination of malaria cases.

Malaria diagnosis in Rwanda is done by using malaria rapid diagnostic tests (RDTs) at the community level and by microscopy or RDTs at the health center level. Data describing the numbers of individuals tested and found to be infected with malaria are reported via the routine HMIS on a monthly basis. Malaria data for the five PBO districts and two standard net districts were extracted for March 2019–February 2022. Monthly positive malaria cases were calculated by adding all confirmed malaria cases reported in the HMIS at the community level (diagnosed by a CHW) and at the health centers, including the inpatient cases from district hospitals. Malaria incidence was calculated using the confirmed malaria cases (a positive diagnostic test for malaria), with the total population counted in 2020 per district as the denominator.

### Statistical data analysis.

Statistical differences in malaria incidence before and after net distribution were determined using the Student’s *t* test. The incidence rate ratio (IRR) was calculated for each of the 2 years post campaign, March 2020–February 2021 and March 2021–February 2022, with the year prior to the campaign (March 2019–February 2020) as the baseline. Statistical analyses were performed in R version 4.2.1 using epitools package version 0.5.10.1.

## RESULTS

### Net distribution and coverage.

Overall, a total of 5,892,280 ITNs were distributed via mass campaigns countrywide with an overall 93.7% coverage. These included 1,085,517 PBO nets distributed in the five PBO districts and 394,013 standard nets distributed in the two control districts. Data on net usage were not collected. No differences were observed in the average net coverage, defined as the number of persons covered per net between the PBO and standard net districts with the average coverage of 2.1 persons per net in the five PBO districts, which was higher than the target of one net for 1.8 people (Rwanda Ministry of Health, 2020) but comparable to the 2.0 persons per net coverage in the standard net districts of Nyaruguru and Nyamagabe (Table 1). A small percentage of nets was also distributed to pregnant women and children via the EPI and ANC routine channels in both the PBO and standard net districts.

**Table 1:** Net distribution per district and distribution channel

Study districts	Population	Mass campaign	EPI and ANC	Total nets	LLIN coverage
Districts with PBO nets					
Kicukiro	392,526	168,550	18,633	187,213	2.1
Gasabo	651,924	302,050	22,699	324,749	2.0
Nyarugenge	349,792	152,400	3,049	155,449	2.3
Rulindo	354,203	161,350	21,063	182,413	1.9
Gicumbi	488,563	212,500	23,193	235,693	2.1
Total	2,237,008	996,850	88,667	1,085,517	2.1
Districts with standard nets					
Nyamagabe	420,094	196,050	21,895	217,945	1.9
Nyaruguru	360,308	156,850	19,218	176,068	2.0
Total	780,402	352,900	41,113	394,013	2.0

ANC = antenatal clinic; EPI = expanded program of immunization; LLIN = long-lasting insecticidal net; PBO = pyrethroid plus piperonyl butoxide.

### Malaria incidence 1 year before and 1 and 2 years after ITN distribution.

One-year post ITN distribution data showed a reduction in total malaria cases in all seven districts (Table 2). However, the percentages of reductions in malaria cases were higher in the PBO districts, with an overall average reduction of 71% compared with the two districts that received standard nets, which had an overall decrease of 51%. Compared with malaria cases reported before the ITN distribution, a sustained reduction (76%) in malaria cases was observed in the PBO districts 2 years after as opposed to the districts that received standard nets, which showed only a marginal decline of 2% as a result of a rebound in malaria cases in these districts 2 years post intervention. Comparable malaria incidence was reported in all seven districts before the ITN distribution (Table 2). One year after the ITN distribution, a significant reduction in malaria incidence was reported in both the PBO (*P* = 0.019) and standard net (*P* = 0.008) districts. However, 2 years after, only the five PBO districts reported a sustained reduction in the incidence of malaria (*P* = 0.013) compared with precampaign levels, whereas the two districts that received standard nets reported similar malaria incidence to the precampaign level (*P* = 0.685) as a result of a rebound in malaria cases (Table 2).

**Table 2:** Malaria incidence 1 year before and 1 and 2 years after ITN distribution

Study districts	Districts	Population (2020)	Malaria cases			Percentage point decline		Malaria incidence per 1,000 persons		
			One year before	One year after	Two years after	One year after	Two years after	One year before	One year after	Two years after
PBO districts	Gasabo	651,924	224,552	66,398	42,699	70	81	344	102	65
	Gicumbi	488,563	58,720	16,746	24,352	71	59	120	34	50
	Kicukiro	392,526	64,721	23,163	21,780	64	66	165	59	55
	Nyarugenge	349,792	40,726	12,910	9,323	68	77	116	37	27
	Rulindo	354,203	56,676	9,998	10,777	82	81	160	28	30
	Overall	2,237,008	445,395	129,215	108,931	71	76	181	52*	46*
Control districts	Nyamagabe	420,094	73,554	38,933	73,449	47	0	175	93	175
	Nyaruguru	360,308	68,397	30,368	65,928	56	4	190	84	183
	Overall	780,402	141,951	69,301	139,377	51	2	182	88*	179

ITN = insecticide-treated net; PBO = pyrethroid plus piperonyl butoxide.

* Significant difference observed in malaria incidence compared with 1 year before ITN distribution levels.

### Malaria incidence rate ratios 1 year before and 1 and 2 years after ITN distribution.

**Figure 2.**
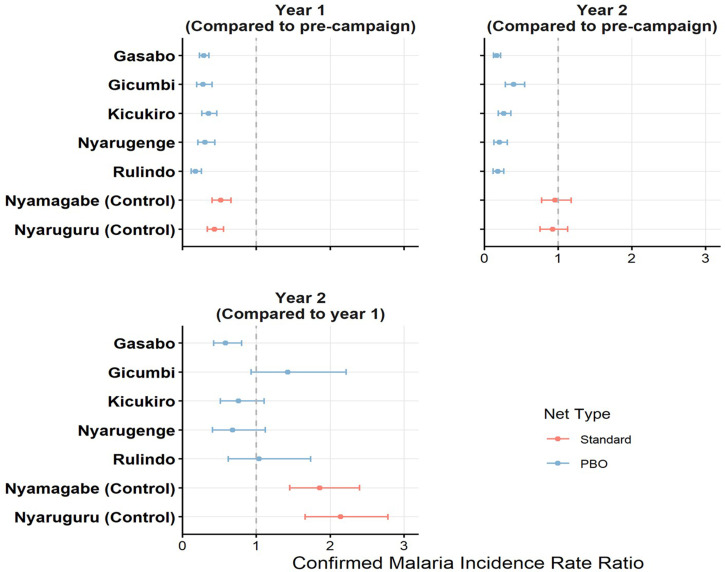
Malaria incidence rate ratios 1 and 2 years after insecticide-treated net distribution. PBO = pyrethroid plus piperonyl butoxide.

Malaria incidence rates were calculated for each district 1 year before and 1 and 2 years after ITN distribution. The IRRs were then calculated using the incidence rate reported 1 year before the distribution as the baseline. The IRRs 1 and 2 years after ITN distribution are shown in Figure 2 and Table 3. The incidence rates 1 year after ITN distribution were lower in all seven districts compared with incidence rates in the year before ITN distribution (IRR < 1). The two control districts that received standard nets showed the lowest decrease (Table 3). In the second year, the malaria incidence rate continued to be significantly lower than the precampaign level in all PBO districts, whereas the burden was the same as the precampaign level in the standard net districts (Table 3). Malaria incidence rate 2 years post ITN distribution compared with the incidence rate in the first-year post ITN distribution was similar in the districts that received PBO nets, except in Gasabo, which had a significantly lower incidence rate (Table 3). However, malaria incidence rate in year 2 significantly increased compared with year 1 post ITN distribution in the standard net districts (Table 3).

**Table 3:** Malaria IRR with 95% CI and *P* values 1 year and 2 years post ITN distribution

Study districts	Year 1 compared with 1 year before ITN distribution		Year 2 compared with 1 year before ITN distribution		Year 2 compared with year 1	
	IRR (95% CI)	*P* value	IRR (95% CI)	*P* value	IRR (95% CI)	*P* value
Gasabo	0.286 (0.229–0.355)	< 2 e-16	0.167 (0.126–0.218)	< 2 e-16	0.583 (0.422–0.799)	0.000736
Gicumbi	0.279 (0.189–0.401)	1.85E-13	0.398 (0.284–0.548)	6.48E-09	1.427 (0.929–2.216)	0.105225
Kicukiro	0.349 (0.259–0.464)	4.08E-14	0.264 (0.188–0.361)	< 2 e-16	0.755 (0.512–1.105)	0.148576
Nyarugenge	0.304 (0.208–0.435)	5.47E-12	0.206 (0.131–0.311)	< 2 e-16	0.677 (0.402–1.121)	0.129918
Rulindo	0.174 (0.115–0.255)	< 2 e-16	0.180 (0.120–0.262)	< 2 e-16	1.034 (0.618–1.733)	0.897422
Nyamagabe (control)	0.514 (0.399–0.657)	7.30E-08	0.956 (0.777–1.177)	0.672921	1.860 (1.451–2.399)	6.83E-07
Nyaruguru (control)	0.432 (0.333–0.555)	1.50E-11	0.924 (0.755–1.130)	0.441647	2.139 (1.659–2.780)	2.08E-09

IRR = incidence rate ratio; ITN = insecticide-treated net.

The incidence relative rates (IRRs) were obtained using malaria incidence rates calculated at year one and year two after ITN distribution in comparison with incidence rates reported at year one before the distribution of nets as the baseline (precampaign) (Table 3). Blue bars represent data obtained in the districts that received PBO nets, whereas red bars represent districts that received standard nets. The small dot represents the IRR and the line whiskers represent the 95% CI. The dashed line indicates an IRR of 1.

## DISCUSSION

As previously reported, data from this study demonstrate that districts that received PBO nets reported a higher reduction of malaria incidence compared with districts that received standard nets during the 2-year study period. The malaria burden was significantly lower within the first year of the campaign, regardless of the net type distributed; however, the benefits of the PBO nets were still observed 2 years after distribution, which was not the case with the districts with standard nets. The five districts that received PBO nets are districts with confirmed resistance to pyrethroids^21^; therefore, as previously reported,^7^^,^^17^^,^^19^^,^^20^ results from this study imply that PBO nets are an effective intervention even in regions in which pyrethroid resistance has been reported. This observation was not caused by differences in the ITN coverage, which was shown to be similar in both PBO and standard net districts. In addition, the malaria transmission in these districts was also similar. However, ITN usage was not investigated, and this could have an impact on the results. Data from the 2017 malaria indicator survey in Rwanda indicated that overall, 64% of household populations in Rwanda slept under an ITN the night before the survey,^23^ and no major differences were observed within the districts.

Other factors known to affect the incidence of malaria include early and effective case management of cases, additional vector control interventions, such as IRS and larviciding, and climatic factors. Case management at the community level plays a critical role in improving the health of populations by extending health care to communities. Currently, in Rwanda, around 58% of all malaria cases nationwide are managed by CHWs deployed in all 14,837 villages from the 30 districts of Rwanda. Indeed, Rwanda has experienced a decrease in malaria incidence and mortality over the last 5 years in part as a result of the country’s community health program established in 1995, characterized by a progressive scale-up of service packages delivered by CHWs. Therefore, it is possible that the observed declines in malaria in this study are also linked to this improved case management of malaria; however, during the observational study period, no changes in case management of malaria were made in any of the study districts and neither was reporting of case methodology different between the two study periods, implying that these factors did not compromise the comparisons made.

A cofounding factor in this study was the fact that in one of the five PBO districts (Gasabo), larval source management using a biolarvicide, *Bacillus thuringiensis var israelensis*, a supplemental vector control intervention, was conducted during the study period. The larviciding was performed at a small scale, via aerial spraying with drones and supplemented by hand applications in an irrigated rice marshland of Gasabo District. This intervention was performed from July 2020 to April 2021 and covered an area of 336 per ha contingent to 15 villages from 4 sectors of Gasabo District (Gatsata, Kinyinya, Jabana, and Gisozi). Despite this additional intervention, we did not observe significant differences in malaria incidence in Gasabo District compared with the other PBO districts that did not have larviciding. However, this could explain the observation that malaria incidence 2 years post ITN distribution compared with the incidence in the first year post ITN distribution was still lower in Gasabo District but not in the other four PBO districts.

Codeployment of ITNs and IRS in the same districts is common practice in some countries in Africa in their attempts to accelerate malaria control progress. This was not the case in the districts investigated. In Rwanda, IRS is typically codeployed with standard ITNs in low- to medium-transmission districts. The five districts that received PBO nets are non-IRS districts and similarly the two districts that received standard nets were not included in the IRS campaign. The decision to deploy PBO nets in these districts was based on confirmed pyrethroid resistance.^21^ Therefore, the data presented point to the effect of these new types of nets in the absence of the well-known malaria-reducing effects of IRS. Previous studies in Rwanda and other countries have demonstrated that malaria incidence is greatly reduced by the use of IRS. It is not known what effect PBO nets would have in similar high-transmission settings, but if a similar reduction is seen to what is observed with IRS, then deployment of PBO nets would provide a much-needed cheaper alternative to IRS. However, studies are required to ascertain this in high-transmission settings in Rwanda.

The negative effects of pyrethroid resistance on the effectiveness of standard ITNs have been less clear and harder to quantify than on IRS.^24^ Although entomological evidence suggests that these nets become less effective at killing mosquitoes under household conditions when high resistance develops,^25^ studies in Benin^26^ and Kenya^27^ demonstrated that pyrethroid-only ITNs remain protective against malaria infection even in areas of moderate insecticide resistance. Additionally, a large WHO-coordinated multicountry study found that these nets protected against malaria despite insecticide resistance.^28^ Several reasons can explain these observations, including the fact that the physical barrier provided by the net might mitigate some of the loss in bioefficacy due to resistance. Nonetheless, standard nets provide only partial protection, and thus integrated vector control strategies and investments in novel tools including next-generation nets are still important.^17^

In this study, standard nets were shown to be less protective than PBO nets 2 years after net distribution, as indicated by the rebound in the incidence of malaria. This could simply imply that the protective effect of standard nets is less than that of PBO nets. Net durability and insecticide content during the 2 years of data collection were not reported in this study. However, previous studies from Rwanda showed that standard net durability and insecticide content are good for less than 2 years,^29^ which could account for the results observed. Suspected but not confirmed pyrethroid resistance was reported in these two districts, and these results could signify that pyrethroid-resistant mosquitoes are in circulation; ongoing entomological monitoring studies will update the malaria program of the current pyrethroid situation in these districts and additional studies will determine the protective impact of PBO nets beyond 2 years.

Ideally, data collected from a randomized controlled study in place of an observational study utilizing routine data would have allowed for a more accurate comparison. Despite this and the other limitations of the current study, the results strongly indicate that PBO nets are a promising tool to combat pyrethroid resistance management and thus for effective and efficient malaria control in Rwanda. Additional studies to determine their protective impact beyond 2 years are required to inform policymakers on the frequency of the distribution–replacement cycle and the selection of the type of nets to deploy.

## Financial Disclosure

We acknowledge the U.S. President’s Malaria Initiative and the Global Fund for funding support.
